# Supplementary material for: Identification of photoperiod-induced specific miRNAs in the adrenal glands of Sunite sheep (Ovis aries)
Source: Front Vet Sci. 2022 Jul 22;9:888207. doi: 10.3389/fvets.2022.888207 (PMC9354845; doi:10.3389/fvets.2022.888207)
Supplement: Supplementary file 1 [file Data_Sheet_1.ZIP › Supplementary file/miRDeep2 file.zip!/miRDeep2 file/Know_Structure/SPLP42A1/oar-mir-30c.pdf]

miRBase precursor : oar-mir-30c
Total read count : 75489
oar-miR-30c read count : 75355
remaining reads : 134

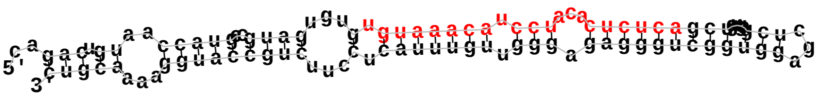

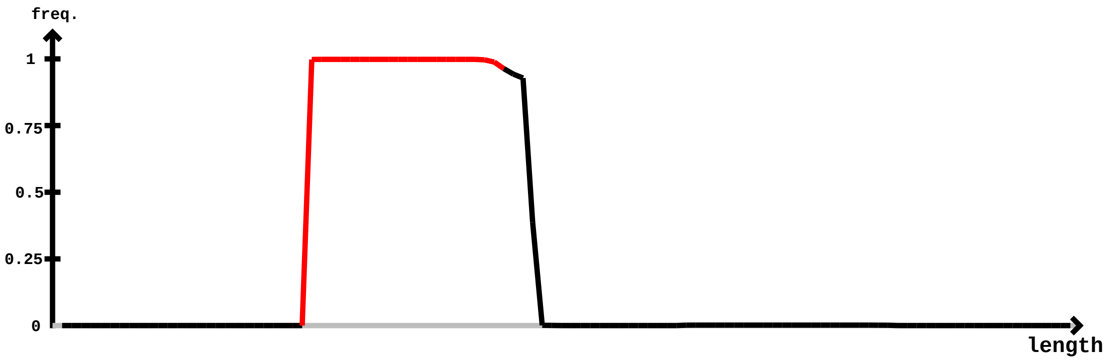


```
                             oar-miR-30c
5'- cagacuguaaccaugccguagugug uguaaacauccuacacucuca gcugugagcucgagguggcugggagaggguuguuuacuccuucugccauggaaaacguc   -3'    exp
    ..(((.((..(((((..((((...(.(((((((.(((...(((((((((....(((...))))))))))))).))).))))))).)...)))))))))...)))))   reads   mm   sample
    cagacuguaaccaugccguagugu.................................................................................   1       0    seq
    ..gacuguaaccaugccguagugug................................................................................   1       0    seq
    .........................uguCaacauccuacacu...............................................................   1       1    seq
    .........................uguaaacauccuacacu...............................................................   3       0    seq
    .........................uguaaacauccuacacuc..............................................................   103     0    seq
    .........................uguaaacauccuacacuU..............................................................   39      1    seq
    .........................uguaaacaCccuacacucu.............................................................   59      1    seq
    .........................uguaaacauccuacacucu.............................................................   555     0    seq
    .........................uguGaacauccuacacucu.............................................................   2       1    seq
    .........................uguaaacauccuacacuUu.............................................................   3       1    seq
    .........................uguaaacaCccuacacucuc............................................................   164     1    seq
    .........................uguaaacauccuacacucuc............................................................   1710    0    seq
    .........................uguaaacauccuacacucuca...........................................................   1437    0    seq
    .........................uguaaacaCccuacacucuca...........................................................   117     1    seq
    .........................uguaaacauccuacacucucag..........................................................   989     0    seq
    .........................uguaaacaCccuacacucucag..........................................................   112     1    seq
    .........................uguaaacaCccuacacucucagc.........................................................   3179    1    seq
    .........................uguaaacauccuacacucucagc.........................................................   37581   0    seq
    .........................uguaaacaCccuacacucucagcu........................................................   1947    1    seq
    .........................uguaaacauccuacacucucagcu........................................................   27248   0    seq
    .........................uguaaacauccuacacucucagcug.......................................................   37      0    seq
    .........................uguaaacaCccuacacucucagcug.......................................................   2       1    seq
    .........................uguaaacauccuacacucucagcugu......................................................   33      0    seq
    .........................uguaaacaCccuacacucucagcugu......................................................   2       1    seq
    ..........................guaaacaCccuacacucuc............................................................   1       1    seq
    ..........................guaaacaCccuacacucucagc.........................................................   2       1    seq
    ..........................guaaacauccuacacucucagc.........................................................   15      0    seq
    ..........................guaaacaCccuacacucucagcu........................................................   2       1    seq
    ..........................guaaacauccuacacucucagcu........................................................   11      0    seq
    ...........................uaaacaCccuacacucucagc.........................................................   1       1    seq
    ................................................................cugggagaggguuguuua.......................   1       0    seq
    ................................................................cugggagaggguuguuuac......................   1       0    seq
    ................................................................cugggagaggguuguuuacu.....................   24      0    seq
    ................................................................cugggagaggguuguuuacuc....................   16      0    seq
    ................................................................cugggagaggguuguuuacucc...................   67      0    seq
```

oar-miR-30c

```
cagacuguaaccaugccguagugugguguaaacauccuacacucucagcugugagcucgagguggcugggagaggguuguuuacuccuucugccauggaaaacguc
................................................................cugggagaggguuguuuacuccu..................  4   0   seq
................................................................cugggagaggguuguuuacuccuu.................  2   0   seq
.................................................................ugggagaggguuguuuacu.....................  2   0   seq
.................................................................ugggagaggguuguuuacuc....................  1   0   seq
.................................................................ugggagaggguuguuuacucc...................  12  0   seq
.................................................................ugggagaggguuguuuacuccu..................  1   0   seq
....................................................................gagaggguuguuuacucc...................  1   0   seq
```
